# Myosin VIIA, Important for Human Auditory Function, Is Necessary for *Drosophila* Auditory Organ Development

**DOI:** 10.1371/journal.pone.0002115

**Published:** 2008-05-07

**Authors:** Sokol V. Todi, Elena Sivan-Loukianova, Julie S. Jacobs, Daniel P. Kiehart, Daniel F. Eberl

**Affiliations:** 1 Neuroscience Graduate Program, The University of Iowa, Iowa City, Iowa, United States of America; 2 Department of Biology, The University of Iowa, Iowa City, Iowa, United States of America; 3 Department of Biology, Duke University, Durham, North Carolina, United States of America; Centre de Regulacio Genomica, Spain

## Abstract

**Background:**

Myosin VIIA (MyoVIIA) is an unconventional myosin necessary for vertebrate audition [1]–[5]. Human auditory transduction occurs in sensory hair cells with a staircase-like arrangement of apical protrusions called stereocilia. In these hair cells, MyoVIIA maintains stereocilia organization [6]. Severe mutations in the *Drosophila* MyoVIIA orthologue, *crinkled* (*ck*), are semi-lethal [7] and lead to deafness by disrupting antennal auditory organ (Johnston's Organ, JO) organization [8]. *ck*/MyoVIIA mutations result in apical detachment of auditory transduction units (scolopidia) from the cuticle that transmits antennal vibrations as mechanical stimuli to JO.

**Principal Findings:**

Using flies expressing GFP-tagged NompA, a protein required for auditory organ organization in *Drosophila*, we examined the role of *ck*/MyoVIIA in JO development and maintenance through confocal microscopy and extracellular electrophysiology. Here we show that *ck*/MyoVIIA is necessary early in the developing antenna for initial apical attachment of the scolopidia to the articulating joint. *ck*/MyoVIIA is also necessary to maintain scolopidial attachment throughout adulthood. Moreover, in the adult JO, *ck*/MyoVIIA genetically interacts with the non-muscle myosin II (through its regulatory light chain protein and the myosin binding subunit of myosin II phosphatase). Such genetic interactions have not previously been observed in scolopidia. These factors are therefore candidates for modulating MyoVIIA activity in vertebrates.

**Conclusions:**

Our findings indicate that MyoVIIA plays evolutionarily conserved roles in auditory organ development and maintenance in invertebrates and vertebrates, enhancing our understanding of auditory organ development and function, as well as providing significant clues for future research.

## Introduction

Mutations in MyoVIIA lead to inner ear transduction anomalies in vertebrates [1], [2], [5]. In humans, MyoVIIA mutations cause syndromic and non-syndromic deafness [3]–[5]. MyoVIIA is expressed by inner ear hair cells [9], [10], where it is important for establishing and maintaining stereocilia organization and for proper auditory transduction [6], [11]. MyoVIIA has been proposed to function in stereocilia cohesion and organization by interacting with a number of proteins [6], [11]–[20], yet its precise function in vertebrate hair cells remains unknown.

**Figure 1:**
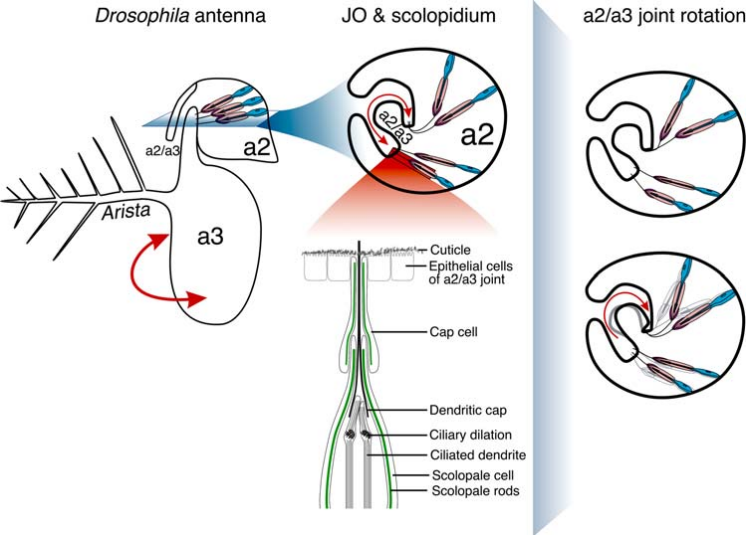
Schematic of the antenna and JO. Drawing not to scale.

The *Drosophila* auditory organ, Johnston's Organ (JO) is evolutionarily related to the vertebrate inner ear [21] and references therein. Auditory transduction in *Drosophila* occurs at the anteriorly placed antennae, each comprising three segments (a1–a3) and a feathery arista. Acoustic stimuli impact the arista to rotate a3 in relation to a2 at the a2/a3 joint, where a3 inserts into a2 (Fig. 1). a2/a3 movements stretch JO mechano-sensitive units (scolopidia) within a2. Each scolopidium consists of neurons with ciliated dendrites and support cells. Ciliated dendrites are enclosed by the scolopale space, an ionically separate area produced by the scolopale cell and supported by actin-rich rods with interspersed microtubules [21]. Dendrites are apically encapsulated by the dendritic cap, an extracellular structure that anchors the scolopidium into the a2/a3 joint [21], [22]. NompA, a putatively filamentous protein secreted by the scolopale cell, is the only dendritic cap component reported to date [22]. The cap cell apically envelopes the scolopale cell and aids in apical attachment (Fig. 1).

*Drosophila* antennae develop from the antennal imaginal discs, clusters of undifferentiated cells in the larva. These discs comprise several concentric folds, the central-most one becoming the distal-most arista and sequentially peripheral ones leading to a3, a2 and a1, respectively [23]. During and after head eversion from the pupal thorax the discs evaginate, increase in size and migrate towards their final position. Neuronal staining with the monoclonal antibody 22C10 indicates that JO is formed from three groups of cells that are first detected in the presumptive a2 a few hours before head eversion [23].

The *Drosophila* orthologue of myoVIIA is encoded by *crinkled* (*ck*) [7]. Previously, we showed that *ck*/MyoVIIA is necessary for adult JO organization and function [8]. Mutations in *ck*/MyoVIIA lead to deafness as a result of scolopidial detachment from the a2/a3 joint. This detachment leads to JO disorganization, most likely due to a *ck*/MyoVIIA role in forming the dendritic cap, which is malformed in mutant flies [8]. Yet, whether *ck*/MyoVIIA is developmentally important for JO organization has not been explored. Since cellular export of the dendritic cap component protein, NompA, appears unaffected by mutations in *ck*/MyoVIIA [8], we used GFP-NompA to follow *ck*/MyoVIIA involvement in JO organization during development. Additionally, we investigated proteins that may affect *ck*/MyoVIIA to elucidate function in the JO by implementing the power of *Drosophila* genetics.

## Results and Discussion

### Johnston's Organ development from the perspective of the dendritic cap

**Figure 2:**
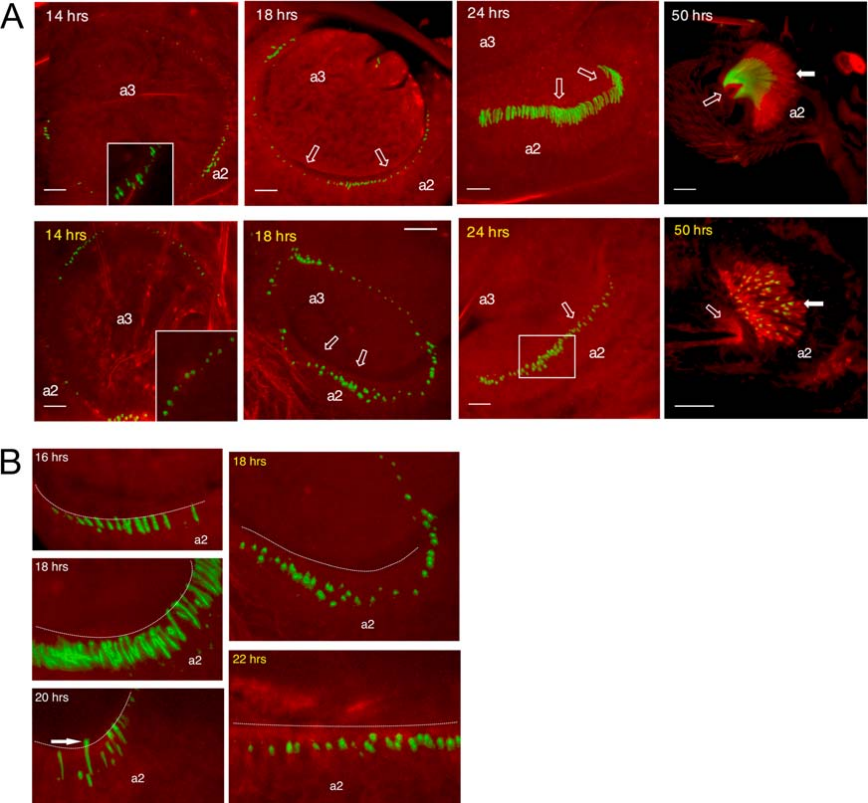
*ck*/MyoVIIA is necessary for JO organization during development. A: Comparison of control (*ck^13^/CyO*; white labels) vs. mutant (*ck^13^/ck^13^*; yellow labels) developing JO. Green channel: GFP-NompA labelling dendritic caps. Red channel: Texas red-phalloidin labelling actin filaments. Block arrows: developing scolopale rods. Open block arrows: direction of a2/a3 boundary. Box indicates disorganization of cap alignment in JO. Inset in 14 hrs control: in some cases the cap shows an elongated profile by this time. Inset in 14 hrs mutant antenna: magnification of the globular profile of the cap. Scale bar = 20 µm. B: Similar staining and labelling as “A”. Note dendritic caps juxtaposed with the perimeter of a2 (dotted line) or extending into the space between a2/a3 in controls (arrow; white labels). In mutants (yellow labels) the caps remain distanced from the future a2/a3 joint.

We focused on JO development after pupal head eversion, which occurs approximately 14 hrs after puparium formation (APF) at 23°C. Dendritic caps were labeled with endogenously expressed GFP-NompA (Fig. 2A). At the time of head eversion, wild type JO is organized as a wide circle; most caps are globular, with few appearing elongated (Fig. 2A, 14 hrs APF and inset). As development progresses, the caps elongate and approach one another (Fig. 2) as the a2/a3 joint constricts. Caps are closely juxtaposed to the space between a2 and a3 as early as 16 hrs APF (Fig. 2B), suggesting that apical connection is already established.

These data indicate that NompA, possibly along with other, unknown cap components, is at least partly produced by the time of head eversion (Fig. 2). As pupation continues, the distance between scolopidia and the future a2/a3 joint increases through the transition of JO from a flat, 2 dimensional disc to a globular, three dimensional organ. During this process, the a2/a3 joint constricts greatly, causing caps to elongate from tension (Fig. 2A).

At 14 hrs APF in wild type animals we observe fewer caps than we do at later stages (e.g. 24 hrs APF, Fig. 2A). This may indicate that not all cells have deposited NompA early in development. Alternatively, scolopidial precursor cell proliferation may be still in progress. Neuronal staining with the monoclonal antibody 22C10 suggested that neuronal numbers increase until approximately 26 hrs APF [23]. Therefore increased cap numbers may indicate further cellular proliferation. A third alternative is that there may be asynchronous activation of GFP-NompA expression.

### *ck*/MyoVIIA is necessary for JO organization during development

We compared developing JO organization in control and *ck^13^* (*ck*/MyoVIIA-null allele [7]) homozygous flies. As evident in Fig. 2A, at the time of head eversion, mutant pupae present a GFP-NompA pattern similar to controls: the caps are aligned in a circle and have a globular profile. But, unlike in controls (Fig. 2A, 14 hrs inset), we saw no caps with an elongated profile in mutant flies. As development continues, caps in *ck*/MyoVIIA-null antennae are in disarray (Fig. 2A). Additionally, while control caps are tightly juxtaposed with, or extending into the future a2/a3 joint in controls (Fig. 2B, 16–20 hrs APF), in mutant pupae, caps retain their globular profile and are distanced from the a2 perimeter (Fig. 2B, 18 and 22 hrs APF). This indicates either that scolopidial apical attachment is not formed in mutants, or that it immediately fails to be maintained.

**Figure 3:**
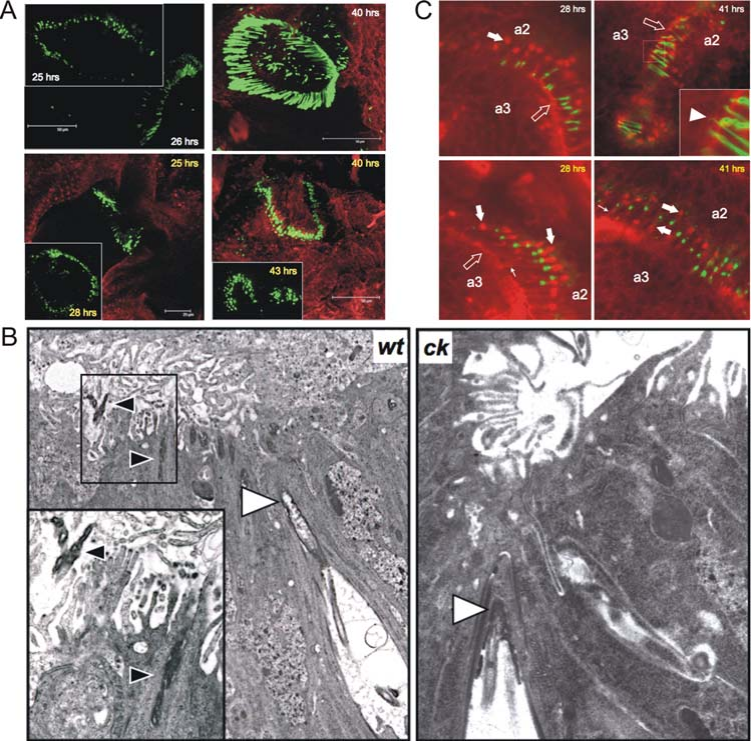
Scolopidia lacking *ck*/MyoVIIA are not attached to the a2/a3 joint during development. A: Wild type (*ck^13^/CyO*; white labels) and mutant (*ck^13^/ck^13^*; yellow labels) cultured antennal discs labelled as in Figure 2 support our finding that *ck*/MyoVIIA is necessary for JO organization. B: Representative EM micrographs from wild type (26 hrs APF) and mutant (28 hrs APF) antennae. Black arrowheads: dendritic caps extending beyond the a2/a3 joint boundary in controls. White arrowheads: dendritic caps at the apical levels of the scolopale space. In *ck* mutants the cap is more compact than in controls. Inset: magnification of the boxed area. C: Wild type (*ck^13^/CyO*; white labels) and mutant (*ck^13^/ck^13^*; yellow labels) cultured antennal discs labelled with anti-cadherin antibody. Green channel: GFP-NompA. Red channel: anti-cadherin. Arrows: cellular junction “tracts” in mutant discs. Open block arrows: a2/a3 boundary. Inset: enlargement of the boxed area. Arrowhead: a dendritic cap extending beyond the a2/a3 boundary. Block arrows: cell-cell junctions.

The data in Figure 2 are supported by findings from cultured antennal discs. Similar to what we observed with developing pupae, *ck*/MyoVIIA is necessary for JO organization from early stages of antennal development in cultured antennal discs. *ck*/MyoVIIA-null antennae have globular, disarrayed dendritic caps, unlike controls (Fig. 3A). We also used electron microscopy (EM) to gain a more detailed perspective on JO development in the presence or absence of *ck*/MyoVIIA (Fig. 3B). At approximately 26 hrs APF, control JOs show dendritic caps clearly extending into the a2/a3 joint (Fig. 3B). Wild-type caps are elongated and slender, compared to mutant JO where we have observed no caps extending into the a2/a3 joint by 28 hrs APF. These data mirror our confocal image analysis (Fig. 2), together indicating that *ck*/MyoVIIA is necessary for early JO organization during development, at least through an involvement at scolopidial apical attachment.

Finally, we labeled control and mutant, cultured antennal discs with an anti-*DE*-cadherin antibody to investigate *ck*/MyoVIIA effects on JO cell-cell junctions during development (Fig. 3C). Cellular junctions, as labeled by this antibody, are present in both control and mutant discs at all developmental stages analyzed. Additionally, we noticed that cell junction “tracts” are visible in JO even though scolopidia are detached in the absence of *ck*/MyoVIIA (Fig. 3C). Together with our observations that another cellular junction marker (β-catenin) localization is unaffected in *ck*/MyoVIIA-null flies (not shown), our data indicate that *ck*/MyoVIIA does not affect the presence of cell-cell junctions in JO.

Our investigations of scolopidial detachment in adult flies [8] and in the developing JO indicate that *ck*/MyoVIIA is important for JO arrangement at a level other than gross cellular morphological juxtapositions. Indeed, our previous data strongly implied dendritic cap anomalies in apical detachment observed in adult flies [8]. We do not know exactly how the dendritic cap is produced, nor the identity of other components besides NompA [21], [22]. Nonetheless, based on the evidence presented here, we can propose a model for the role of *ck*/MyoVIIA in JO organization during development: the dendritic cap is deposited around the dendrites and extends between epithelial cells into the future a2/a3 joint upon cuticle secretion by epidermal cells (Fig. 1). As scolopidium distance from the a2/a3 joint increases during development, the dendritic cap, which has already established its apical attachment, elongates under tension. Scolopale rods grow from assembled actin and may also play a role in this distancing process and cap elongation (in fact, we do not clearly observe scolopale rods using actin labeling until approximately 24 hrs APF, although defects in GFP-NompA distribution are already detected at 18 hrs APF (Fig. 2)). In *ck*/MyoVIIA mutants, as scolopidial distancing from the a2/a3 joint begins, the cap detaches from an inadequate apical anchor, possibly because of dendritic cap morphological anomalies [8] due to faulty transport/deposition of some component, while cell-cell junctions are present among scolopidial components (Fig. 2C and data not shown). This is also supported by our previous investigations of the a2/a3 joint in adult *ck*/MyoVIIA-null JO, where we noticed that a2/a3 epithelial cells remain attached to the joint, and that scolopidial cell-cell junction integrity is intact [8]. Two non-mutually exclusive mechanisms can account for this model. In the first possibility, *ck*/MyoVIIA directly facilitates extension of the dendritic cap through the epithelial layer. Failure of the cap to penetrate through this epithelium prevents the anchoring which is necessary for its sufficient elongation to reach the luminal space for anchoring in the cuticle. In the second possibility, elongation of the cap is purely a passive secondary consequence of tension that requires *ck*/MyoVIIA-mediated anchoring. Thus these two mechanisms differ in whether *ck*/MyoVIIA directly mediates the cap extension or directly mediates the anchoring. It is possible that both mechanisms are operating, but our current data cannot distinguish them. In conclusion, our explorations indicate that *ck*/MyoVIIA functions in JO development in its organization during development, from the perspective of NompA. Initially, the absence of this motor protein leads to faulty apical anchoring, which in turn, leads to scolopidial detachment.

### *ck*/MyoVIIA maintains adult JO organization

**Figure 4:**
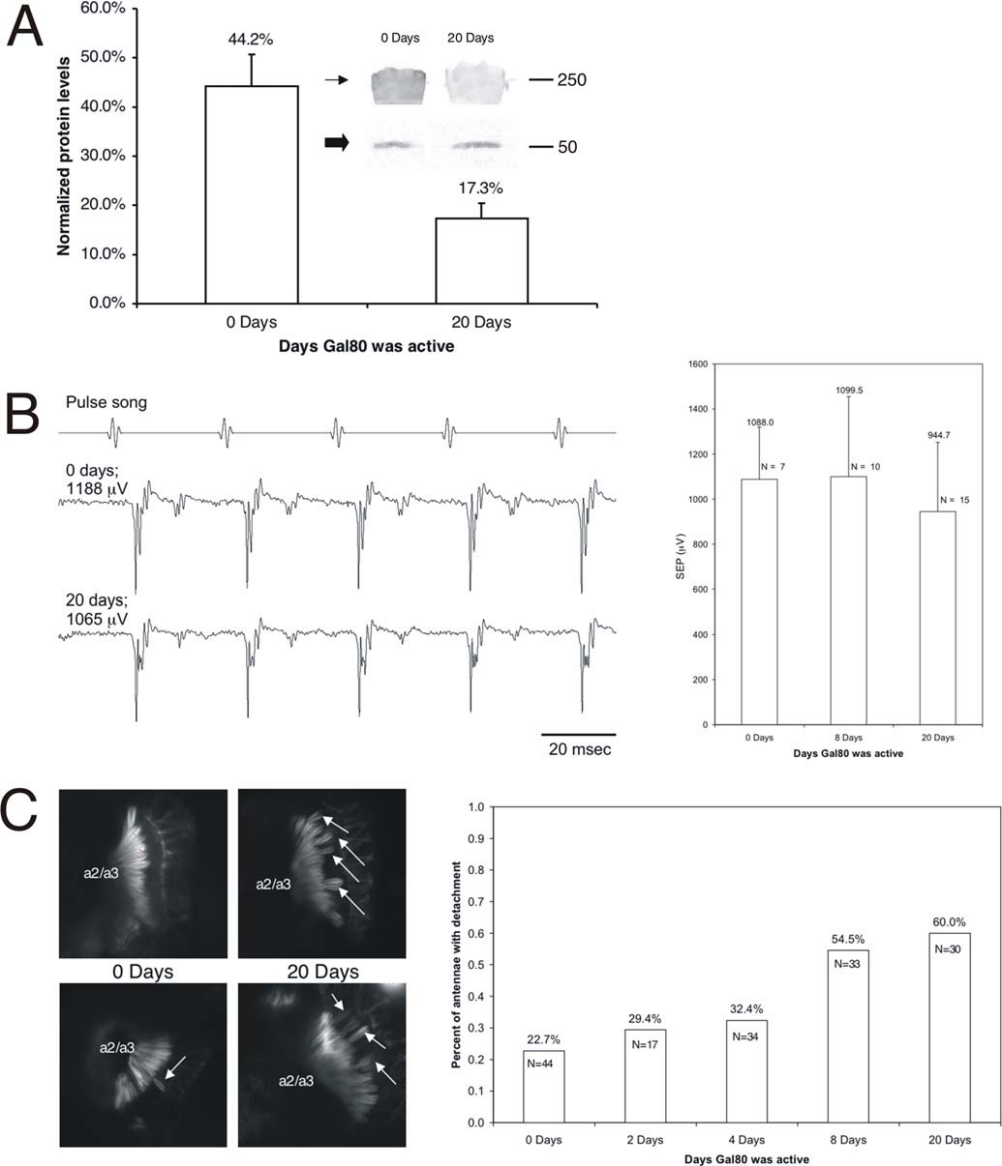
*ck*/MyoVIIA is important for maintaining adult JO organization. A: Histogram of densitometry analysis of the western blot (inset) staining for *ck*/MyoVIIA (arrow) and β-tubulin (block arrow; loading control) from flies with active Gal80 for 0 or 20 days. Mean +/− SD; N = 4. B: Representative electrophysiological recordings (left) from the antennal nerve of flies with active Gal80 for 0, 8 or 20 days show no significant difference. Right: histograms of recordings. Mean +/− SD. C: JO organization in adults. Arrows indicate detachment. Histograms show percent of antennae with detached scolopidia.

Next, we investigated *ck*/MyoVIIA involvement in maintaining adult JO organization. To reduce *ck*/MyoVIIA levels in adult flies we used an ubiquitously-expressed, temperature-sensitive *tubulin-Gal80^ts^* construct [24] to inhibit protein expression using the *Gal4-UAS* system. Active Gal80^ts^ (hereon referred to as Gal80) binds Gal4 and prevents it from activating UAS sequences. Flies maintained at the Gal80 restrictive temperature, on the other hand, express Gal4 target genes. We generated *ck*/MyoVIIA-null adult flies that were fully rescued by a ubiquitously expressed *UAS-GPF-ck* (*GFP-ck*) rescue construct [8], which were also homozygous for *Gal80*. These flies were reared at 29°C (Gal80 restrictive temperature; *GFP-ck*/MyoVIIA expressed) until eclosion, at which time they were transferred to 18°C (Gal80 permissive temperature) for 0–20 days. Under these conditions further expression of *GFP-ck*/MyoVIIA is blocked and the level of GFP-*ck*/MyoVIIA decreases (Fig. 4A). JO is fully mature and functional at eclosion.

We had previously shown that there is a correlation between degree of detachment and degree of deafness associated with *ck*/MyoVIIA mutations when comparing antennae with no, partial, or complete JO organization [8]. To investigate effects of reduced *ck*/MyoVIIA levels (Fig. 4A) on JO transduction, and to gain some insight into JO disorganization through this technique, we performed electrophysiological recordings from the antennal nerve. Recordings from flies maintained at the Gal80 permissive temperature for a period of 0, 8 or 20 days showed no statistically significant difference, indicating that lower *ck*/MyoVIIA levels in these adult flies have no effect on JO transduction (Fig. 4B).

Nevertheless, we used confocal laser microscopy to investigate the extent of detachment present in JO. It is impossible for us to accurately quantify the number of scolopidia detached in a particular JO. However, we could easily provide a semi-quantitative score of no, some or large/full detachment. We compared antennae from flies maintained for 0 days at the Gal80-permissive temperature to ones which were maintained at the Gal80-permissive temperature for up to 20 days. As shown in Fig. 4C, lower *ck*/MyoVIIA levels for increasing periods of time lead to higher numbers of antennae with detached scolopidia. Since we know that aging does not lead to scolopidial detachment (ESL & DFE, unpublished observations) we conclude that *ck*/MyoVIIA is important for maintaining adult JO organization.

Comparing our electrophysiology data with the confocal images, it is likely that a large number of scolopidia need to be detached in order for an electrophysiological difference to be detected with our extracellular recording. Therefore, confocal laser microscopy may be more informative on some finer details of JO organization than is extracellular electrophysiology.

Our data strongly suggest that *ck*/MyoVIIA participates in JO organization through a role in scolopidial apical attachment. Interestingly, similar to *myoVIIA*-deficient mice where stereocilia are initially formed but they become progressively more disorganized [6], *ck^13^* pupae show overall JO organization akin to controls early in development (Fig. 2A; 14 hrs APF). Later, disorganization becomes increasingly evident in the developing JO (Fig. 2A), reminiscent of the large disarray in stereocilia observed in developing vertebrate ears [6]. This supports the idea that MyoVIIA plays evolutionarily conserved functions in the organization of developing auditory organs, although the exact mechanisms through which it acts in organizing them may be different. In JO, *ck*/MyoVIIA is important for proper dendritic cap morphology [8]. It is likely that *ck*/MyoVIIA-related cap malformations are responsible for apical detachment during development and in adult flies. Recent reports from vertebrate studies presented evidence that MyoVIIA is important for proper localization of a number of membrane-associated proteins involved in trans-stereocilia links, including the ankle-link protein Vlgr1, the transmembrane proteins Usherin and Protocadherin 15, and PDZ domain-containing protein Whirlin [12]–[15], [18]–[20]. These mislocalizations may explain the disorganization phenotype observed in MyoVIIA-deficient mice [6]. The identity of proteins for whose transportation *ck*/MyoVIIA is required in JO remains unknown. Whether the transmembrane and submembrane proteins putatively transported by vertebrate MyoVIIA have orthologues in *Drosophila* and whether they could be implicated in JO organization needs to be investigated. Future work employing proteomic approaches, the versatility of *Drosophila* genetics and confocal imaging alongside additional JO cellular markers, will provide further details into the function of this protein necessary for auditory transduction in vertebrates and flies.

### *ck*/MyoVIIA function in JO is modulated by myosin-associated proteins

To understand mechanisms in which *ck*/MyoVIIA participates in JO, we focused on a pathway important to Planar Cell Polarity (PCP; the two-dimensional polarity established within an epithelium). Manifestations of PCP are very obvious in the arrangement and orientation of the stereocilia in the hair cells of the vertebrate inner ear sensory epithelium, as well as in bristle organ and even non-sensory hair organization in insects [25]. While there is no obvious morphological manifestation of PCP associated with JO, it is conceivable that the molecular mechanisms of PCP may operate and could underlie the defects we see in *ck*/MyoVIIA-deficient scolopidia. To address this possibility, we studied a genetic pathway with which *ck*/MyoVIIA interacts in the PCP of the fly wing [26]. Here, each epidermal cell constructs a single prehair, consisting of bundled actin filaments that mature into a single hair that faithfully projects from the distal vertex of each cell [25], [27]. Many studies have examined the mechanisms that govern the molecular and morphological orientation of hairs in these cells.

**Figure 5:**
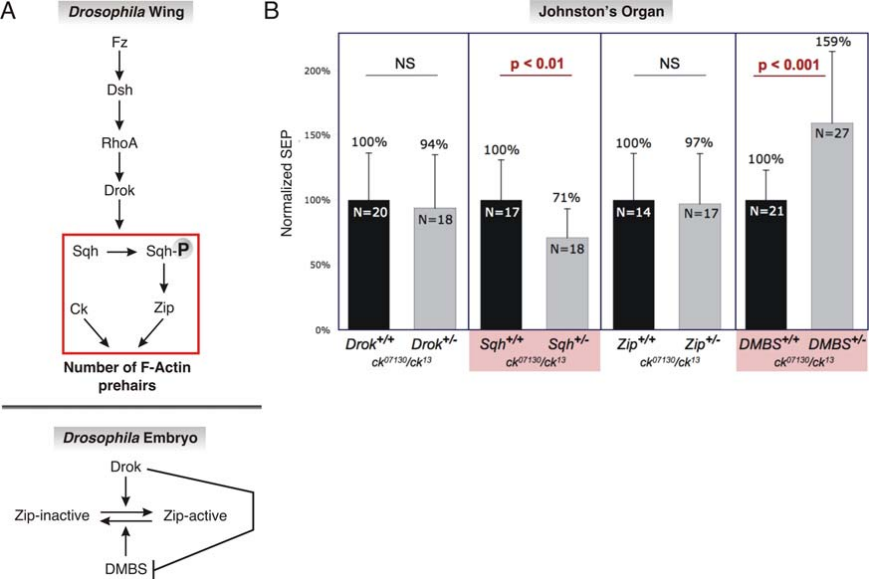
*ck*/MyoVIIA function is modulated by Sqh and DMBS in adult JO. A: Genetic interactions of *ck*/MyoVIIA with actin pathways in the *Drosophila* wing (top panel) and in embryonic dorsal closure (bottom panel). Boxed area indicates the portion of the pathway investigated in panel B. Based on [26], [36]. B: *ck*/MyoVIIA genetically interacts with Sqh and DMBS in adult JO. Responses from flies with only one functional copy of *Drok*, *sqh*, *zip*/MyoII or *DMBS* were normalized to their respective, two-copy sibling controls, all in a sensitized *ck*/MyoVIIA background. Histograms show mean +/− SD.

Frizzled (Fz) a transmembrane receptor of Wnt signals, and Dishevelled (Dsh), an adaptor protein downstream of Fz, are evolutionarily conserved components of PCP [25], [28], [29]. Ligand-dependent activation of Fz leads to Dsh activation, which triggers Drok (*Drosophila* Rho-kinase) (Fig. 5A); in turn, Drok phosphorylates Spaghetti-Squash (Sqh; the nonmuscle myosin II regulatory light chain protein) [26], [30]. Regulatory light chain phosphorylation causes a conformational change in non-muscle myosin II (*zip*/MyoII) that activates *zip*/MyoII motility in *in vitro* motility assays [26], [31], [32]. Throughout phylogeny, myosin II molecules are heterohexamers that consist of a pair of myosin II heavy chains (in flies encoded by *zipper*, *zip*/MyoII), a pair of regulatory light chains (in flies encoded by *spaghetti squash*, *sqh*) and a second pair of so called essential light chains (in flies, encoded by *Mlc-c*, see [33] and references therein). In all systems studied to date, binding of the light chains to the heavy chain is so tight that dissociation constants have not been measured. In the wing, malfunction of any one of the elements in this pathway disturbs the assembly of F-actin prehairs and leads to the aberrant generation of multiple hairs on each cell (Fig. 5A), which are usually interpreted as a defect in PCP or its output.

Interestingly, in the wing there is a strong genetic interaction of *ck*/MyoVIIA with the *fz*-*dsh*-*Drok*-*sqh*-*zip* pathway, where *ck*/MyoVIIA mutations have the opposite effect of *zip*/MyoII heavy chain mutations [26] (Fig. 5A). The molecular basis of this interaction is not known. Analysis of nonmuscle myosin II subunits indicates that in embryos *ck*/MyoVIIA does not bind to Sqh [33]. *zip*/MyoII function is also inhibited by myosin phosphatase which dephosphorylates Sqh at the Drok-dependent sites (Fig. 5A) [34]–[37].

We explored genetic interactions between *ck*/MyoVIIA and *Drok*, *sqh*, *zip*/MyoII and the myosin binding subunit of myosin phosphatase (*DMBS*) in JO by reducing the copy number of each putative interacting gene in a sensitized *ck* background (*ck^13^/ck^07130^*), and by using electrophysiology to determine the effects such reductions had on already compromised auditory transduction [8]. We first focused on *Drok*, *sqh* and *zip*/MyoII genetic interactions with *ck*/MyoVIIA in JO. As evident from histograms in Figure 5B, neither *Drok^1^* (lethal recessive) nor *zip^1^* (lethal recessive) heterozygosity altered transduction ability of *ck*/MyoVIIA hypomorphs significantly (P>0.5). However, *sqh^AX39^* (lethal recessive) significantly reduced sound-evoked potentials (Fig. 5B; P<0.01), suggesting that Sqh normally promotes *ck*/MyoVIIA function in JO scolopidia. Genetic interaction with *sqh* but not *Drok* or *zip*/MyoII was unexpected based on the current understanding of the wing PCP pathway (Fig. 5A). Therefore, we explored whether *ck*/MyoVIIA genetically interacts with DMBS. We generated flies heterozygous for *DMBS^03802^* (lethal recessive), again in a *ck^13^*/*ck^07130^* background. In the presence of only one functional gene copy of *DMBS*, *ck^13^*/*ck^07130^* flies showed a significantly enhanced ability to transduce the pulse song (P<0.001; Fig. 5B) suggesting that DMBS normally suppresses *ck*/MyoVIIA activity in JO. This DMBS interaction is predictably opposite to the interaction with *sqh*, but the direction of both of these interactions is unexpected if their effects are mediated through the same underlying mechanisms as PCP in the wing or embryonic dorsal closure.

These differences could be attributable to two main possibilities. The first is that scolopidial detachment in *ck*/MyoVIIA is due to defects in the PCP pathway outlined in the wing, yet this pathway is skewed in JO, either because: a) some players in the pathway are absent, b) new players are present, or c) levels of players are shifted sufficiently in a tissue-dependent manner. The second possibility is that scolopidial detachment is due to functions of *ck*/MyoVIIA independent of a role in PCP. While we currently cannot distinguish formally between these two possibilities, the overall profile of genetic interactions presented here may favor the PCP-independent mechanisms. Interestingly, the role of MyoVIIA in vertebrate hearing has not been directly associated with a core role in PCP, despite the highly oriented arrangement of hair cells that clearly rely on PCP mechanisms for their precision.

In summary, we have demonstrated that *ck*/MyoVIIA is acutely required early in the apical attachment of scolopidia, either by directly facilitating dendritic cap extension through the epithelial layer, or anchoring the cap during tensioning and elongation. Furthermore, we have shown a continuing requirement for *ck*/MyoVIIA to maintain attachments throughout adulthood. Finally, we have presented evidence that in the JO, *ck*/MyoVIIA genetically interacts with the non-muscle myosin light chain regulatory protein (Sqh) and the myosin binding subunit of myosin phosphatase (DMBS). Our data suggest that the mechanism of *ck*/MyoVIIA in JO differs from a previously characterized PCP pathway (Fig. 5A), at least at the level of the components we examined (Drok, *zip*/MyoII; Fig. 5B). Instead, novel genetic interactions between *ck*/MyoVIIA and myosin regulatory proteins appear to operate in JO. Whether these tissue-specific interactions reflect deployment of alternative forms of PCP or whether the JO function of *ck*/MyoVIIA is unrelated to PCP functions remains to be established. Our data are consistent with the idea that similar genetic pathways may affect auditory transduction in vertebrates, and draw attention to the complexity of myosin regulatory mechanisms in different cell types that still require dissection in *Drosophila* and vertebrate models of deafness.

## Methods

### Fly strains

*ck^13^* is described in [7]. *GFP-NompA* flies were from M. Kernan (SUNY Stony Brook). *DMBS^03802^* flies were from N. Perrimon (Harvard Medical School). *Drok^1^* and *sqh^AX39^* flies were from L. Luo (Stanford University). All pairs of genotypes tested arose as siblings from the same cross.

### Electrophysiology

Electrophysiology was performed as previously described [38]. Error bars indicate standard deviation.

### Pupal dissection

Pupae, maintained at 23°C, were timed from the moment of puparium formation. The pupal case was removed with forceps at both ends and the everted head was exposed. Pupae were fixed overnight in 4% paraformaldehyde (in PBS).

### Antennal cultures

Cultures of eye-antennal discs from animals at the white prepupal stage were established as previously described [39], [40]. Schneider's *Drosophila* medium (GIBCO), supplemented with 2–4% heat-inactivated fetal bovine serum (GIBCO), 1% penicillin/streptomycin (GIBCO) and 20-hydroxyecdysone (0.2 µg/mL; Sigma-Aldrich) was used.

### Immunohistochemistry, EM and confocal microscopy

Procedures were performed as previously described [8]; anti-*DE*-cadherin antibody was DCAD2 (rat) from the University of Iowa Developmental Studies Hybridoma Bank, used at 1∶20 in blocking solution, overnight. Other antibodies and materials were used as previously described [8].

### Western blotting

Flies were collected at eclosion, maintained at 18°C for 0 or 20 days and frozen at −80°C. 4 males per group (0 or 20 days) were homogenized in 100 µL hot sample buffer (0.125 M Tris pH 6.8, 1% SDS, 1 mM EDTA, 10% sucrose, 1% BME) for 20 seconds, boiled for 5 minutes and spun briefly. Samples were loaded into a 7% polyacrylamide gel, transferred onto PVDF membrane and blocked for 1.5 hours in 5% dry milk∶TBS-Tween solution. The membrane was incubated overnight at 4°C in 1∶1000 primary antibody∶blocking solution (anti-*ck*/MyoVIIA [7], or anti-β tubulin, University of Iowa Developmental Studies Hybridoma Bank). The membrane was rinsed four times for 5 minutes each with blocking solution, incubated in donkey anti-guinea pig (*ck*/MyoVIIA) or donkey anti-mouse (tubulin) HRP-conjugated secondary antibody (Jackson Labs; 1∶1000 in blocking solution) for 50 minutes, rinsed three times for 5 minutes each with TBST, then once for 5 minutes in TBS and developed with a peroxidase substrate kit (Vector).

### Gal80 confocal examination

Flies were reared in a 29°C-regulated environment to inhibit Gal80 activity until they eclosed. They were transferred to an 18°C-regulated environment and maintained there for 0–20 days. Antennae were dissected, stained with Texas Red- or Oregon Green-phalloidin and prepared for confocal microscopy as previously described [8].

### Statistics

Statistical analyses were performed using two-tailed, Student T-tests.

## References

[1] Ernest S, Rauch G-J, Haffter P, Geisler R, Petit C (2000). *Mariner* is defective in *myosin VIIA*: a zebrafish model for human hereditary deafness.. Hum Mol Genet.

[2] Gibson F, Walsh J, Mburu P, Varela A, Brown KA (1995). A type VII myosin encoded by the mouse deafness gene *shaker-1*.. Nature.

[3] Liu X-Z, Walsh J, Mburu P, Kendrick-Jones J, Cope MJTV (1997). Mutations in the myosin VIIA gene cause non-syndromic recessive deafness.. Nature Genet.

[4] Liu X-Z, Walsh J, Tamagawa Y, Kitamura K, Nishizawa M (1997). Autosomal dominant non-syndromic deafness caused by a mutation in the myosin VIIA gene.. Nature Genet.

[5] Weil D, Blanchard S, Kaplan J, Guilford P, Gibson F (1995). Defective myosin VIIA gene responsible for Usher syndrome type 1B.. Nature.

[6] Self T, Mahoney M, Fleming J, Walsh J, Brown SDM (1998). Shaker-1 mutations reveal roles for myosin VIIA in both development and function of cochlear hair cells.. Development.

[7] Kiehart DP, Franke JD, Chee MK, Montague RA, Chen TL (2004). Drosophila *crinkled*, mutations of which disrupt morphogenesis and cause lethality, encodes fly myosin VIIA.. Genetics.

[8] Todi SV, Franke JD, Kiehart DP, Eberl DF (2005). Myosin VIIA defects, which underlie the Usher 1B Syndrome in humans, lead to deafness in *Drosophila*.. Curr Biol.

[9] Hasson T, Gillespie PG, Garcia JA, MacDonald RB, Zhao Y-d (1997). Unconventional myosins in inner-ear sensory epithelia.. J Cell Biol.

[10] Hasson T, Heintzelman MB, Santos-Sacchi J, Corey DP, Mooseker MS (1995). Expression in cochlea and retina of myosin VIIa, the gene product defective in Usher syndrome type 1B.. Proc Natl Acad Sci (USA).

[11] Kros CJ, Marcotti W, van Netten SM, Self TJ, Libby RT (2002). Reduced climbing and increased slipping adaptation in cochlear hair cells of mice with *Myo7a* mutations.. Nature Neurosci.

[12] Adato A, Lefèvre G, Delprat B, Michel V, Michalski N (2005). Usherin, the defective protein in Usher syndrome type IIA, is likely to be a component of interstereocilia ankle links in the inner ear sensory cells.. Hum Mol Genet.

[13] Adato A, Michel V, Kikkawa Y, Reiners J, Alagramam KN (2005). Interactions in the network of Usher syndrome type 1 proteins.. Hum Mol Genet.

[14] Boëda B, El-Amraoui A, Bahloul A, Goodyear R, Daviet L (2002). Myosin VIIa, harmonin and cadherin 23, three Usher I gene products that cooperate to shape the sensory hair cell bundle.. EMBO J.

[15] Delprat B, Michel V, Goodyear R, Yamasaki Y, Michalski N (2005). Myosin XVa and whirlin, two deafness gene products required for hair bundle growth, are located at the stereociliary tips and interact directly.. Hum Mol Genet.

[16] El-Amraoui A, Petit C (2005). Usher I syndrome: unravelling the mechanisms that underlie the cohesion of the growing hair bundle in inner ear sensory cells.. J Cell Sci.

[17] Küssel-Andermann P, El-Amraoui A, Safieddine S, Hardelin J-P, Nouaille S (2000). Unconventional myosin VIIA is a novel A-kinase-anchoring protein.. J Biol Chem.

[18] Küssel-Andermann P, El-Amraoui A, Safieddine S, Nouaille S, Perfettini I (2000). Vezatin, a novel transmembrane protein, bridges myosin VIIA to the cadherin-catenins complex.. EMBO J.

[19] Michalski N, Michel V, Bahloul A, Lefèvre G, Barral J (2007). Molecular characterization of the ankle-link complex in cochlear hair cells and its role in the hair bundle functioning.. J Neurosci.

[20] Senften M, Schwander M, Kazmierczak P, Lillo C, Shin J-B (2006). Physical and functional interaction between protocadherin 15 and myosin VIIa in mechanosensory hair cells.. J Neurosci.

[21] Todi SV, Sharma Y, Eberl DF (2004). Anatomical and molecular design of the *Drosophila* antenna as a flagellar auditory organ.. Microsc Res Techn.

[22] Chung YD, Zhu J, Han Y-G, Kernan MJ (2001). *nompA* encodes a PNS-specific, ZP domain protein required to connect mechanosensory dendrites to sensory structures.. Neuron.

[23] Lienhard MC, Stocker RF (1991). The development of the sensory neuron pattern in the antennal disc of wild-type and mutant (*lz^3^*, *ss^a^*) *Drosophila melanogaster*.. Development.

[24] McGuire SE, Le PT, Osborn AJ, Matsumoto K, Davis RL (2003). Spatiotemporal rescue of memory dysfunction in *Drosophila*.. Science.

[25] Mlodzik M (1999). Planar polarity in the Drosophila eye: a multifaceted view of signaling specificity and cross-talk.. EMBO J.

[26] Winter CG, Wang B, Ballew A, Royou A, Karess R (2001). *Drosophila* Rho-associated kinase (Drok) links Frizzled-mediated planar cell polarity signaling to the actin cytoskeleton.. Cell.

[27] Wong LL, Adler PN (1993). Tissue polarity genes of Drosophila regulate the subcellular location for prehair initiation in pupal wing cells.. J Cell Biol.

[28] Krasnow RE, Wong LL, Adler PN (1995). Dishevelled is a component of the frizzled signaling pathway in Drosophila.. Development.

[29] Sokol S (2000). A role for Wnts in morpho-genesis and tissue polarity.. Nature Cell Biol.

[30] Mizuno T, Amano M, Kaibuchi K, Nishida Y (1999). Identification and characterization of *Drosophila* homolog of Rho-kinase.. Gene.

[31] Tan JL, Ravid S, Spudich JA (1992). Control of nonmuscle myosins by phosphorylation.. Annu Rev Biochem.

[32] Chrzanowska-Wodnicka M, Burridge K (1996). Rho-stimulated contractility drives the formation of stress fibers and focal adhesions.. J Cell Biol.

[33] Franke JD, Boury AL, Gerald NJ, Kiehart DP (2006). Native nonmuscle myosin II stability and light chain binding in Drosophila melanogaster.. Cell Motil Cytoskel.

[34] Alessi D, MacDougall LK, Sola MM, Ikebe M, Cohen P (1992). The control of protein phosphatase-1 by targetting subunits. The major myosin phosphatase in avian smooth muscle is a novel form of protein phosphatase-1.. Eur J Biochem.

[35] Hartshorne DJ, Ito M, Erdodi F (1998). Myosin light chain phosphatase: subunit composition, interactions and regulation.. J Muscle Res Cell Motil.

[36] Mizuno T, Tsutsui K, Nishida Y (2002). Drosophila myosin phosphatase and its role in dorsal closure.. Development.

[37] Tan C, Stronach B, Perrimon N (2003). Roles of myosin phosphatase during *Drosophila* development.. Development.

[38] Eberl DF, Hardy RW, Kernan M (2000). Genetically similar transduction mechanisms for touch and hearing in *Drosophila*.. J Neuroscience.

[39] Milner MJ, Haynie JL (1979). Fusion of *Drosophila* eye-antennal imaginal discs during differentiation in vitro.. Roux's Arch Dev Biol.

[40] Li C, Meinertzhagen IA (1995). Conditions for the primary culture of eye imaginal discs from *Drosophila melanogaster*.. J Neurobiol.

